# *“Self arranged Cactis”* as new goethite morphology from the natural corrosion process of SAE 1020 carbon steel

**DOI:** 10.1016/j.heliyon.2019.e02771

**Published:** 2019-11-27

**Authors:** Thiago G. Costa, Vanessa Wandersee Cunha Ostroski, Fernando S. de Souza

**Affiliations:** a Laboratory of Materials, Atelier for the Conservation-Restoration of Movable Cultural Heritage, Fundação Catarinense de Cultura (Santa Catarina Culture Foundation), 88025-200, Florianópolis, SC, Brazil; b UNISOCIESC, SociedadeEducacional de Santa Catarina, Chemical Eengineering Departament, 89206001, Joinville, SAC, Brazil

**Keywords:** Electrochemistry, Inorganic chemistry, Materials chemistry, Natural corrosion, Electron microscopy, Morphology, Goethite, Carbon steel

## Abstract

A new morphology of goethite aggregates (α-FeOOH) obtained through the natural corrosion process of 1020 carbon steel parts exposed to weathering was found. Micrographies obtained by SEM reveal micro and nanostructures with forms of nanosquares, microparticles, nanowires inside microparticles and the unpublished structure of "*Self arranged Cactis*", all varying between 115 nm and 8 μm. The molecular structure of goethite was characterized by FTIR and elemental analysis of EDX converged with the obtained data. The average corrosion rate for 1020 carbon steel in the weathering was 1.7592 mpy. The data obtained in this work will contribute to the understanding of the corrosion process of 1020 carbon steel, one of the most used in civil construction, as well as in material sciences, where iron oxides are widely used in metallurgy, catalysis and adsorption, and the domain of morphology is fundamental for each application.

## 1 Introduction

The understanding and characterization of metallic oxides resulting from the aging process of iron alloys has undeniable importance for several areas of knowledge, with emphasis on physics, chemistry and materials science, its multidimensional structures are fundamental to correlate with its applications and diagnosis of pathologies [1, 2, 3]. In this way, the iron metal oxides have potential applications in the areas of catalysis [4], metallurgy [5], adsorption [6] and still important in the understanding of the degradation process of iron-based cultural art works [7]. In this sense, the morphological study of the nano and micro materials formed by these oxides, which may present in the Magnetite (Fe_3_O_4_), Maghemita (γ-Fe_2_O_3_), Haematite (α-Fe_2_O_3_) and Goethite (α-FeOOH) as the spectroscopic analyzes are fundamental for their correct characterizations and applications.

Several authors report the synthesis of nano and microparticles of iron oxides using different precursors with the intention of modeling their forms and influencing their morphologies, especially nano rods, wires, cubes, rings and spidles [1, 2, 8, 9, 10]. In recent work, Sayed and Polshettiwar [11] reported the preparation of nano iron oxide particles using various iron salts as precursors and various experimental conditions to obtain different morphologies depending on the precursor and reaction medium. However, natural corrosion products of iron-based materials are poorly studied at the micro- and nanostructural level, and these are of fundamental chemical importance for the understanding of the aging process of materials used in different technological areas from the art works until construction.

In the present work, a way for obtaining solid oxide-hydroxide of iron in the form of Goethite α-FeOOH with new morphology was employed from samples of 1020 carbon steel exposed to weathering that underwent a natural corrosion process. This iron corrosion product was characterized by infrared spectroscopy - FTIR, the morphology elucidated by scanning electron microscopy - SEM and elemental analysis performed by X - ray dispersive energy - EDX microanalysis.

## 2 Materials & Methods

### 2.1 Preparation of the samples

Fifteen 1020 carbon steel test samples were used for atmospheric weathering tests with dimensions of 100 × 70 mm, with a total area of 7000 mm^2^.

### 2.2 Loss of mass by weathering

The technique of weathering (ABNT NBR 6209) [12] was used to determine the loss of mass suffered by the carbon steel 1020, proceeded by the calculation of the corrosion rate. The samples were fixed to avoid contact and overlapping of the neighboring pieces in an exhibition shelf, which was made in wood with dimensions of 1,25 m of height and 0,65 m of width, guaranteeing the adequate rigidity of the specimens, with mechanical stability to withstand the force of the winds and the mass of the 18 sample tests, giving exposure to the lower part of the samples. The place of exposure for the atmospheric test was to the sea front of the São Francisco do Sul city in Santa Catarina State, near the Port of São Francisco do Sul, where there is a bulk cargo operation, 10 m from the sea, with the face facing the continent. As standard, the exposure angle was 60° in relation to the horizontal, meeting international standards and requirements ASTM D 1014 [13] and ABNT NBR 6209/2007. The triplicate samples were taken at the following periodicities: 14, 27, 41 and 50 days. Afterwards, they were thoroughly cleaned of the corrosion products in a Clark solution (1.0 L 37% HCl, 20.0 g of antimony (III) chloride (SbCl_3_) and 50.0 g of tin(II) chloride (SnCl_2_)). Every 5 min the samples were removed from the bath and washed in running water. This process was repeated repeatedly until the variation of the sample weight was no more significant in relation to the total weight of the sample. The corrosion rate was calculated by following equation derivate from Faraday equation:

$$Corrosion\;rate\;\left(CR\right)=\;\frac{K.W}{A.t.d}\;;$$

where:

K = proportionality constant to adequate the unities = 8,76 x 10^4^
W = loss of mass = final mass (g) - initial mass (g)
A = sample exposure area (cm^2^)
t = exposure time (h)
d = sample density (g cm^3^)

### 2.3 Scanning electron microscopy with energy dispersive X-Ray spectroscopy – SEM-EDS

The morphologies of each fragment were obtained by micrography using a scanning electron microscope model Phenom Prox-X with 1000–22000 of magnification. The EDS spectra were collected in a spectrometer by dispersive energy of X-rays connected to the scanning electron microscope using a 15 kV accelerating voltage.

### 2.4 Infrared spectroscopy - FTIR

The infrared spectra were collected in the tree samples; the spectrometer JASCO, a model FTIR-4100 with 4 cm^−1^ resolution was used. The samples were homogenized in KBr pastilles in which approximately 3 mg of each for analysis, and all spectra were collected with 64 scans.

## 3 Results and discussion

### 3.1 Loss of mass by weathering

Observing the tendency of mass loss of the SAE 1020 carbon steel sample exposed to weathering, it is possible to note that the corrosion product is soluble, but little adherent to the surface of the samples. Thus, the accumulation of corrosion products leads to a decrease in the exposed area in the corrosive medium over time. This fact is in agreement with the expected, because iron oxides are removed from the surface of the metal because of the action of rain, wind and dew. However, the large amount of corrosion products formed along the exposure of the specimens causes the products to accumulate on the surface of the samples and the decrease of the exposure area occurs.

Using an exposure area of 70 cm^2^ (7000 mm^2^) it was possible to calculate the corrosion rate as a function of time loss and subsequent classification in terms of atmospheric corrosivity found at the Port of São Francisco do Sul, according to the NACE RP-07-75 standard [14]. The values of analysis parameters are shown in Table 1.

**Table 1:** Mean values of mass loss found in 1020 carbon steel samples exposed to weathering.

Exposure time (h)	Initial mass (g)	Final mass (g)	Weight loss (g)	Corrosion rate (mm year^−1^)	Classification as a function of corrosion rate
336	81.2977	80.9997	0.2980	0.1412	C3–C4
648	84.4716	84.1714	0.3002	0.0738	C3–C4
816	82.6255	82.3240	0.3015	0.0588	C3–C4
1200	81.8260	81.5148	0.3112	0.0413	C3–C4

It was observed with the weathering test in the port environment that initially the corrosive process is intense, and its average speed decreased with the time of exposure. After the corrosion product has formed, the material will corrode at a rate which depends on the composition of the material and the corrosion product, in this case the iron oxides, which over time agglomerate on the surface of the metal material. However, the iron oxides layer is porous, and this makes it difficult to adhere to the steel surface, and the marine environment is classified as medium to high atmospheric corrosivity.

### 3.2 Infrared spectroscopy - FTIR

**Fig. 1:**
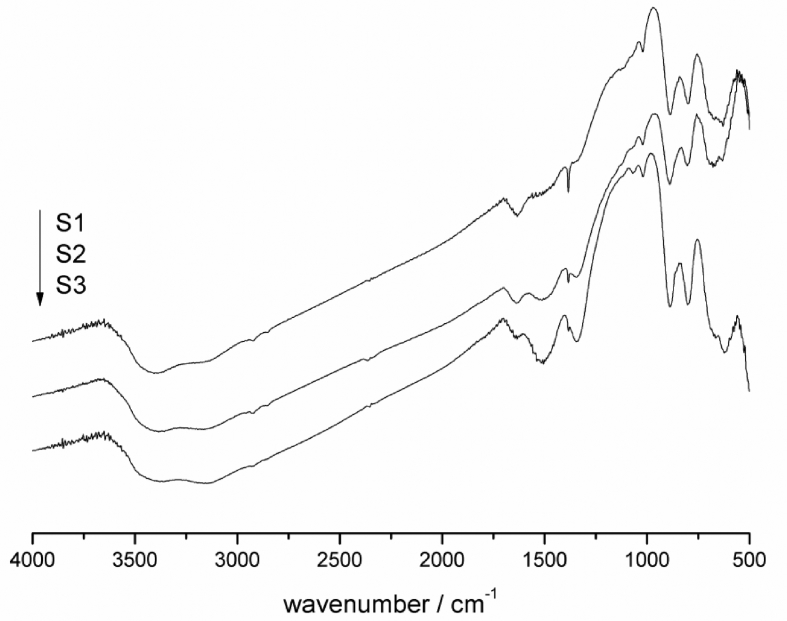
FTIR spectra of the degradation products of 1020 carbon steel samples.

The FTIR analyzes are widely used in the characterization of iron oxides and hydroxides, the FTIR spectra of three different patina sites observed in the exposed samples can be observed in Fig. 1. It can be noted that all the spectra are very similar, and present bands at 889 and 797cm^−1^ assigned to (δ Fe–O–H) bending vibrations and 625cm^−1^ attributed to (ν Fe–O) stretching vibrations [15, 16]. The vibrational modes attributed to the hydroxyl bending region between 1300-1700cm^−1^ [17], in our case 1632 and 1510cm^−1^ (δ O–H) and (γ O–H) are observed.

The bands observed at 2900-3500 cm^−1^ can be characterized by stretching of hydroxyl group. The described stretches are characteristic of the vibrational modes associated with the goethite structure (α-FeOOH), confirming the molecular structure of the major corrosion product, in this way the oxidation product of the 1020 weathered carbon steel samples is formed by goethite.

**Scheme1:**
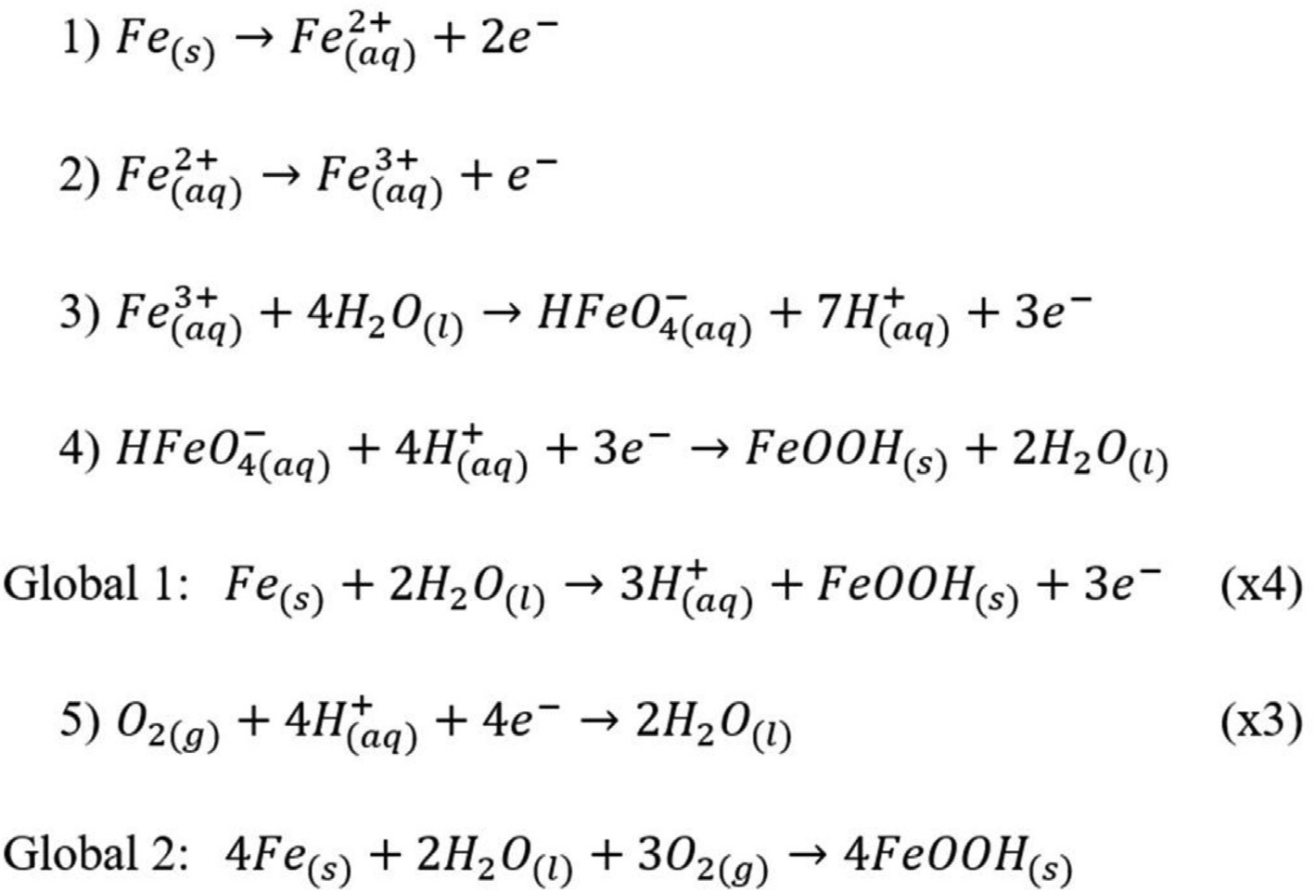
Electrochemistry semi-reactions and global reactions for goethite product formation by the oxidation of carbon steel under experimental weathering conditions.

The formation of the goethite structure (α-FeOOH) under the described experimental conditions can be explained by the electrochemical reactions describe in Scheme 1.

In this propose, Scheme 1, the semi-reaction 1 presents the oxidation of metallic iron to iron(II), in the partial oxidation state (II). In the following, the semi-reaction 2 indicate the formation of iron(III) from iron(II) previously formed. In the semi-reaction 3, the formation of acid iron(VI) oxide in the presence of water (moisture). The HFeO_4_^-^ ion is the predecessor of the iron(III) oxide-hydroxide.

The semi-reaction 4 shows the reduction of the iron specie from (VI) to (III), leading to the formation of the species of interest (FeOOH). Until this stage, only the iron species are mentioned, and Global Reaction 1 indicates the partial balance of the equations involving the iron species. Semi-reaction 5 indicates the participation of oxygen in the overall process, which Global Reaction 2 was obtained for the corrosion process of iron alloy exposed to weathering (presence of water and oxygen).

### 3.3 Scanning electron microscopy with energy dispersive X-Ray spectroscopy – SEM-EDS

The morphologies of inorganic materials in micro and nanometric scale are great importance for several areas in the materials science and technology fields. In this sense, knowing the microscope of oxidation products from iron base products is extremely important to understand the processes of corrosion of base materials of this metal, that is great interest for an industry as well as for a conservation of art works and cultural goods that use this support - many cultural artifacts are submerged today and the understanding of morphology is essential for the diagnosis of their conservation status as well as for characterization of materials exposed in the experimental conditions of this work. Several works approach the experimental methods and the different types of morphology obtanied from different iron precursors [18, 19, 20, 21, 22, 23, 24].

**Fig. 2:**
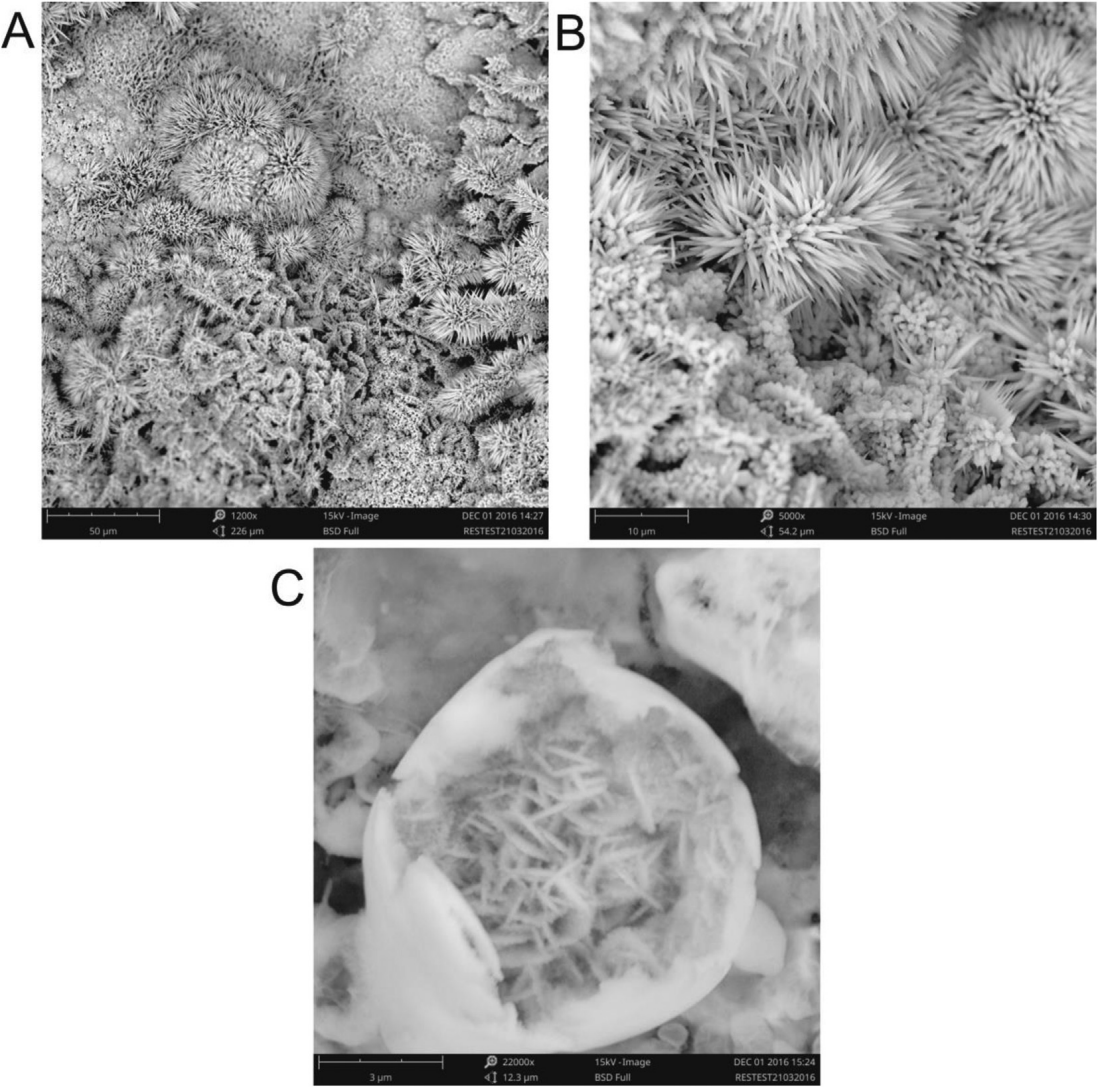
Micrographs with magnification between 1200–22000x of degradation products of 1020 carbon steel samples. Were (A) Self arranged “Cactis”, (B) Nanosquares and Self arranged “Cactis” and (C) Microparticles and Nanowires inside microparticles.

Through SEM analysis, Fig. 2, three types of morphology are included that call "*self-organized Cactis*", nanosquas and nanowires within microparticles of 1020 carbon steel oxidation products can be observed [11]. Note the micrographs that nanosquas, which measure about 420 nm in width, form a base type for the " self-arranged Cactis", which have the average diameter of 381 nm. Also, it is noted that in some larger ones such as 22000x there are as microparticles with 8mm diameter and within the delimitations can be characterized by another typical morphology that are nanowires, which are about 115nm in diameter. Table 2 presents a description of each morphology as well as its average sizes.

**Table 2:** Description of the morphologies and particle size.

Morphology (Shape)	Size (average)
Self arranged “Cactis”	381nm (diameter)
Nanosquares	420nm
Nanowires inside microparticles	115nm (diameter)
Microparticles	8 μm (diameter)

Recently, Sayed and Polshettiwar [11] reported a simplified and sustainable method for the synthesis of iron oxide nanoparticles and evaluated the effect of iron salts as precursors on the morphology of the products. The authors reported six morphological forms obtained synthetically varying between nanorods up to self arranged flowers, with respect to the goethites no data were found referring to one of the morphologies obtained in this work, " self arranged Cactis", which shows us a new microscopic structure found in natural conditions, where even corrosion studies under similar experimental conditions, did not lead to the obtaining of this microstructure [25].

**Fig. 3:**
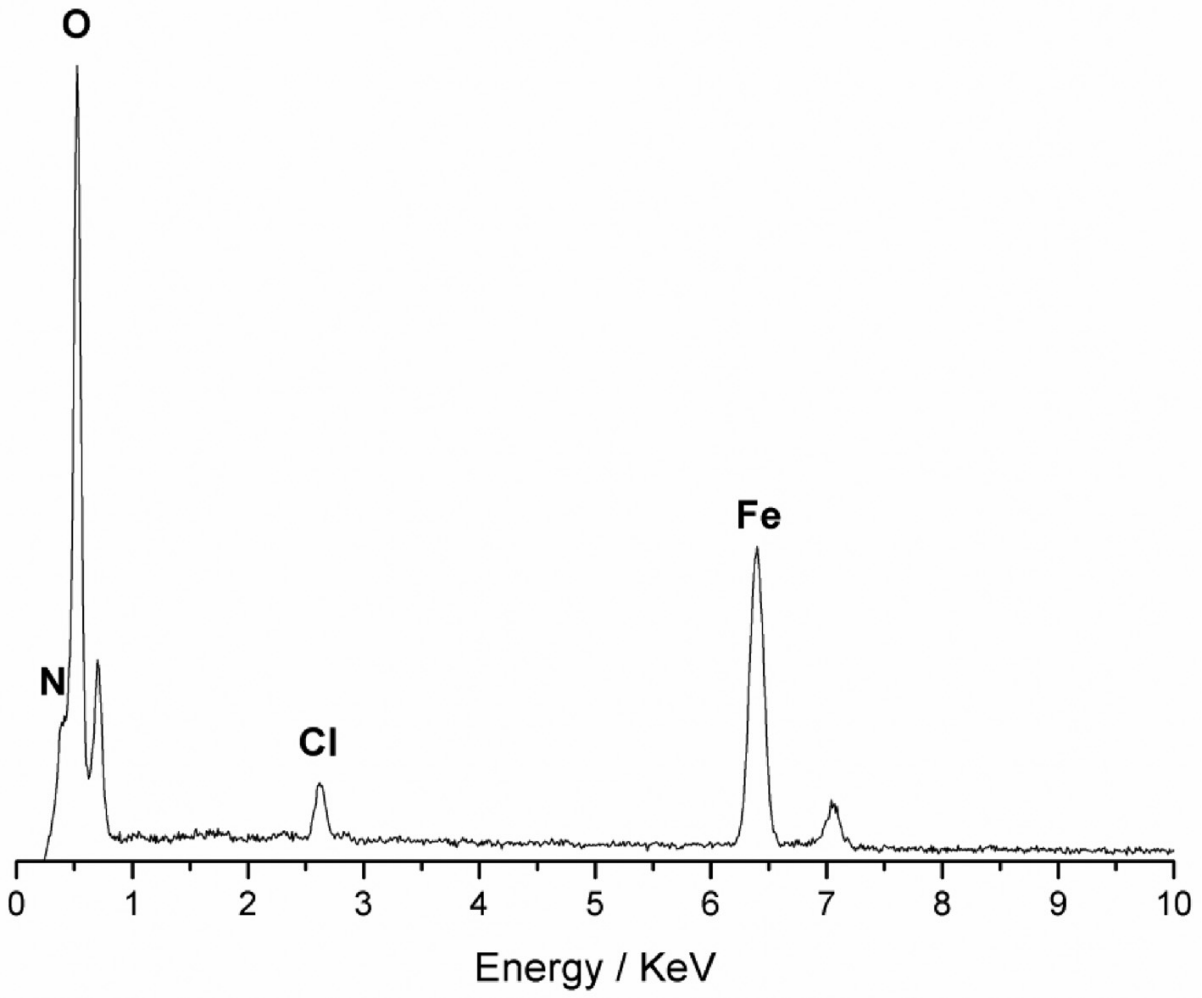
Typical EDX spectrum found in several areas of 1020 steel carbon degradation products.

The SEM- EDS technique is also widely used in the elemental characterization of iron oxides from various sources, whether synthetic or natural, and found extensively in recent works [26, 27, 28, 29, 30, 31, 32]. The EDX spectra obtained in several regions of the degraded products of the weathered steel follow the pattern shown in Fig. 3. In all the spectra the major elements Fe, Cl, O and N are observed, with iron and oxygen converge to the molecular structure characterized by FTIR of goethite, related to the oxidation product of the iron present in the metal alloy. The chlorine and nitrogen is associated with minority compounds, probably iron chlorides, in the environment that the specimens have been exposed to, and nitrogen may be in inorganic form (nitrates and nitrites) or organic (compounds with nitrogen functions). However, the elements found in lesser amounts are not associated with the goethite matrix characterized by SEM-EDX. Table 3 shows the elemental quantities found by the EDX technique, and the results also converge to the structure characterized by vibrational spectroscopy.

**Table 3:** Elemental composition found by ESD of 1020 steel carbon degradation products.

Element	Weight percentage
Fe	41 ± 0.14
O	51.25 ± 0.3535
Cl	2.35 ± 0.0701
N	5.4 ± 0.14

## 4 Conclusions

In this work, natural corrosion products of 1020 carbon steel were obtained by natural weathering, characterized by spectroscopic methods and their micro and nanometric structure elucidated by SEM. The major chemical composition obtained in the final products was goethite (α-FeOOH), an oxide-hydroxide of iron. In relation to morphology, an unpublished form of organization of these aggregates, called "*Self arranged Cactis*", was found. This discovery will help researchers and professionals from different areas in the application of these materials, as well as in the identification of patinas in structures and objects based on carbon steel and encourage studies on oxides and different materials based on iron, searching for new structures and new applications.

## Declarations

### Author contribution statement

Thiago G. Costa: Performed the experiments; Analyzed and interpreted the data; Contributed reagents, materials, analysis tools or data; Wrote the paper.

Vanessa Wandersee Cunha Ostroski: Conceived and designed the experiments; Analyzed and interpreted the data; Contributed reagents, materials, analysis tools or data.

Fernando S. de Souza: Conceived and designed the experiments; Performed the experiments; Analyzed and interpreted the data; Contributed reagents, materials, analysis tools or data; Wrote the paper.

### Funding statement

This research did not receive any specific grant from funding agencies in the public, commercial, or not-for-profit sectors.

### Competing interest statement

The authors declare no conflict of interest.

### Additional information

No additional information is available for this paper.No additional information is available for this paper.
